# Emotion Regulation in Dementia Caregiving: The Role of Neuropsychiatric Symptoms and Attachment Orientation

**DOI:** 10.1177/08919887231195228

**Published:** 2023-08-04

**Authors:** Tânia Brandão, Rute Brites, João Hipólito, Odete Nunes, Catarina Tomé Pires

**Affiliations:** 1 William James Center for Research, 56068 Ispa – Instituto Universitário, Lisboa, Portugal; 2 CIP-UAL, Departamento de Psicologia, 70992 Universidade Autónoma de Lisboa Luís de Camões, Lisboa, Portugal

**Keywords:** dementia, neuropsychiatric symptoms, attachment, emotion regulation

## Abstract

Family caregivers are usually the main source of support for persons living with dementia, being exposed to a loved one’s suffering, which can lead to experiencing strong and negative emotions. This study aimed to identify factors capable of explaining individual differences in the way caregivers regulate their emotions. This cross-sectional study included 78 informal caregivers (M = 64.84 years; SD = 13.32) and 84 controls (non-caregivers) (M = 77 years; SD = 7.59). Neuropsychiatric symptoms (NPS), attachment orientations, and emotion regulation were measured using self-report scales. Caregivers of persons living with dementia used more expressive suppression in comparison to non-caregivers. NPS and attachment avoidance were associated with expressive suppression. Moderation analyses showed that NPS only predicted expressive suppression when attachment avoidance was low or medium. The present study showed that caregivers are more likely to suppress their emotions in the presence of NPS, especially those with lower/middle levels of attachment avoidance. Psychological interventions targeting emotion regulation should be offered especially to caregivers that face NPS of persons living with dementia and present lower/middle levels of attachment avoidance.

## Introduction

Dementia can be overwhelming for both the persons living with dementia and their main caregiver. Literature has shown that providing care to a close one with a chronic condition, such as dementia, can have deleterious consequences for the caregivers. Specifically, studies have shown that informal caregivers of persons living with dementia, usually a relative or close family member, are more likely to report psychological distress and psychopathological symptoms in comparison to non-caregivers.^1,2^ Additionally, informal caregivers of persons living with dementia tend to experience higher levels of burden and burnout,^3^ especially when the care is provided by the spouses since they usually report even more physical, psychological, and financial difficulties than other caregivers.^4^

These difficulties felt by informal caregivers of persons living with dementia can be explained by several reasons. Most studies have focused on the role played by disease-related factors (eg, neuropsychiatric symptoms (NPS), lower autonomy, fewer medications) or sociodemographic characteristics of the caregiver (eg, sex or age).^5^ However, less attention has been paid to the fact that caregivers are usually exposed to a loved one’s suffering which can lead to experiencing strong and negative emotions such as distress, anger, frustration, guilt, or helplessness.^6^ Thus, informal caregivers of persons living with dementia need to continuously regulate these emotions that arise from the perceptions of care recipient suffering making emotion regulation (ER) - which refers to “the processes by which individuals influence which emotions they have, when they have them and how they experience and express them”, ^7^(p. 275) - an important factor to be examined within this context.

Two main strategies of ER have been examined in psychological research: cognitive reappraisal that refers to individuals’ efforts to change the meaning of a stimulus to alter its emotional impact. It is an antecedent-focused strategy since it occurs early in the emotion-generative process (ie, before the generation of an emotional response). And expressive suppression that refers to individuals’ efforts to hide or inhibit an emotion-expressive behaviour. It is a response-focused strategy since it takes place late in the emotion-generative process (ie, when an emotional response was already generated), being a form of response modulation.^8-10^ Thus, while cognitive reappraisal has been linked to more positive and less negative affect as well as better well-being and interpersonal functioning (eg, in terms of social support, close relationships, and likability), expressive suppression has been linked to more negative affect and poor well-being, and poor interpersonal functioning.^8-11^

A closer look to the literature about ER on the context of caregiving reveals that this research topic has been rarely explored. The few available studies with caregivers have suggested that engaging in cognitive reappraisal and emotion expression is associated with less reactivity in caregiving spouses,^12,13^ while others have pointed to the detrimental effects of expressive suppression for caregivers.^14^ Also, expressive suppression is likely to be linked to individuals’ poor well-being as well as to lower relationship quality within dyads (as found by some authors in the context of parental caregiving).^15^

To the best of our knowledge, no previous studies have examined potential differences ER strategies between individuals who are caregivers and those who are not. However, it would be expected that caregivers would have more difficulties in regulating their emotions (eg, use more expressive suppression and less cognitive reappraisal) due to the chronic and high-stress nature of caregiving. For instance, they may feel pressure to maintain a positive attitude and not burden the care recipient with their own emotions.

In 1 study, exploring the role of emotion suppression of caregivers taking care of persons with traumatic brain injuries,^16^ the authors concluded that caregivers assume that they are forced to suppress their emotions for maintaining the appearance that they are emotionally adapted to. Also, they may suppress their emotions as way to avoid feeling overwhelmed or to avoid the stigma from others about their caregiving role. The chronic and high-stress nature of caregiving can also impair their abilities to use cognitive reappraisal since it is a cognitively demanding strategy.^8-10^

In this study, we aimed to contribute to better understand individual differences in the way caregivers of persons living with dementia regulate their emotions, namely in terms of cognitive reappraisal and expressive suppression. Two variables will be examined as potential factors influencing ER: NPS and attachment orientations.

### The Role of Neuropsychiatric Symptoms

Caregiving in the context of dementia disease is particularly difficult and challenging due to the presence of behavioural and psychological symptoms in persons living with dementia such as agitation, depression, apathy, repetitive questioning, psychosis, aggression, sleep problems, and wandering, among other socially inappropriate behaviours.^17,18^ As pointed out by Kiles et al,^17^ these symptoms are complex and stressful being linked to poor patients’ health outcomes as well as to poor caregivers’ psychological functioning. Several studies have explored the role played by NPS on the psychological functioning of caregivers of persons living with dementia. They have been associated with more depression and higher caregiving burden, distress, and burnout.^19-23^

No studies, however, have explored the role played by NPS in explaining individual differences in the way caregivers regulate their emotions. Yet, some studies suggested that deficits or declines in some emotion behaviours of persons living with dementia (eg, visual avoidance, lack of empathy, difficulties in ER) may indeed contribute to influencing the psychological functioning of the caregivers.^24,25^ Because ER is an interpersonal process,^26^ we believe that these changes can also contribute to influencing caregivers’ ER due to a lack of reciprocity in interactions between the members of the couple.

### The Role of Attachment

Attachment theory is a useful framework for understanding interpersonal dynamics in the context of dementia caregiving since it offers an approach to understanding the dynamics of caring for others who are in suffering through the activation of the caregiving system.^27,28^ Additionally, it allows us to understand ER processes.^29^

Attachment refers to a significant and intense emotional bond with a significant other.^30^ Attachment orientations, that derive from internal working models (ie, mental representations about the self, others, and relationships that are developed based on early life experiences with primary caregivers), shape and guide individuals’ behaviours and feelings.^31^ Overall, more securely attached individuals are more likely to provide comfort and support while more insecurely attached individuals are less comfortable or competent to provide support, avoiding the situation or providing a type of support that is not adequate.^32^

Also, attachment orientations contribute to explaining individual differences in ER processes. More securely attached individuals tend to use more adaptive ER strategies (eg, cognitive reappraisal) allowing them to cope with stress, while more avoidantly attached individuals tend to rely on deactivating strategies (eg, suppression) and more anxiously attached individuals on hyperactivating strategies (eg, rumination) that tend to amplify the stress experienced rather than help them to cope with it.^29,33,34^

In the context of dementia caregiving, the activation of the caregiving system allows individuals to provide care for a relative living with dementia that is (becoming) dependent and needs support. Some studies suggested that attachment orientations are likely to influence the NPS of persons living with dementia. For instance, Perren et al^35^ found that higher levels of caregivers’ avoidance were associated with higher levels of problem behaviours in persons living with dementia (especially agitation/aggression). Also, Monin et al^28^ found that more anxiously attached persons living with Alzheimer were more likely to report more physical and psychological symptoms, especially when their caregivers were also more anxiously attached. For these reasons, we believe that NPS and attachment style may contribute to explaining individual differences in the way caregivers regulate their emotions, both separately and in interaction. Collectively, the findings of these studies suggest that individuals with an insecure attachment orientation, particularly caregivers, may be prone to employing expressive suppression in response to NPS due to their heightened difficulty in coping with stressful situations, greater likelihood of using maladaptive ER strategies, and more negative evaluations of these symptoms, which may intensify perceived threats or cause a distancing from problematic relationships.^27-29,33,34^ Also, caregivers with an insecure attachment style may have a more strained and conflictual relationship with their care recipient, which could exacerbate the impact of NPS on their emotional regulation.^35^

### The Current Investigation

The aims of this study were: (1) to examine whether informal caregivers of persons living with dementia and non-caregivers differ in the use of 2 specific strategies of ER: cognitive reappraisal and expressive suppression; (2) to explore the role of NPS and attachment orientations as variables capable of explaining differences in the use of ER strategies in the informal caregiver sample.

Previous studies showed that caregivers experience more psychological distress and psychopathological symptoms in comparison to non-caregivers.^1,2^ Also, studies showed that ER influences individuals’ psychological functioning.^8-11^ And in some contexts,^16^ caregivers assume that they are forced to suppress their emotions for maintaining the appearance that they are emotionally adapted to. For these reasons, we hypothesized that caregivers would use more expressive suppression and less cognitive reappraisal in comparison to non-caregivers (H1) due to role demands or lack of support.

Additionally, we hypothesized that NPS and attachment orientations would contribute (solely and in interaction) to explaining differences in ER in informal caregivers of persons living with dementia. Previous studies have not explored the role of NPS in explaining ER differences. Some authors have suggested that deficits or declines in some emotion behaviours of persons living with dementia^24,25^ may contribute to influencing the psychological functioning of the caregivers also in terms of ER because these deficits may limit interpersonal dynamics that are important for emotion regulatory processes. Attachment orientations have been linked to ER.^29,33,34^ Thus, we hypothesized that attachment avoidance would be associated with more suppression (to maintain control, minimize proximity, or avoid vulnerability) and less cognitive reappraisal (due to their tendency to dismiss threatening thoughts, limiting cognitive engagement); and that attachment anxiety would be associated with less suppression and less cognitive reappraisal (due to the cognitive inflexibility to reframe events) (H2).^34^

Finally, we hypothesized that caregivers’ attachment would moderate the association between NPS and ER. There is evidence from some studies that suggests a link between insecure attachment styles, including those of caregivers, and increased levels of problem behaviours related to dementia.^28,35^ Additionally, as found in previous studies,^29,33,34^ individuals with higher levels of avoidance or anxiety have more difficulties in ER, being more likely to suppress their emotions and less likely to engage in cognitive reappraisal. Thus, we hypothesized that NPS would be associated with more suppression and less cognitive reappraisal only for those with higher levels of avoidance and lower levels of anxiety (ie, those with an insecure attachment) (H3).

## Method

### Participants

Persons living with a medical diagnosis of dementia and living in the community were included in the study. Informal caregivers were eligible if they were the primary caregiver of the patient and had no payment or training for caregiving. Caregivers were excluded if they had previous or current participation in individual or group psychotherapy for addressing caregiving-related issues, suffered from severe chronic disease or psychiatric disorder, or had a history of drug or alcohol abuse (as self-reported).

The control group (non-caregivers) consisted of adults from the community without caregiving responsibilities. To be eligible, participants must not have had a severe chronic disease or psychiatric disorder and not have a history of drug or alcohol abuse (as self-reported).

### Measures

#### Emotion Regulation

The ERQ-Emotion Regulation Questionnaire was used to examine expressive suppression and cognitive reappraisal.^9^ This 10-item self-report scale assesses expressive suppression (4 items, item example: “I control my emotions by not expressing them”) and cognitive reappraisal (6 items, item example: “I control my emotions by changing the way I think about the situation I’m in”). Items are rated on a 7-point Likert scale (1 = “strongly disagree” to 7 = “strongly agree”). In the current study, both dimensions presented good internal consistency values (α = .75 for expressive suppression; α = .82 for cognitive reappraisal).

#### Neuropsychiatric Symptoms

The Neuropsychiatric Inventory (NPI) evaluated the presence of NPS in persons living with dementia.^36^ It is composed of questions on 12 domains: delirium, hallucinations, agitation, depression/dysphoria, anxiety, euphoria/relation, apathy/indifference, disinhibition, irritability/lability, motor behaviour, sleep, and appetite, each assessed with 1 question.^37^

Respondents are asked to refer to the previous month to indicate the presence of these symptoms in a yes/no format question.^36^ When they answer yes, they should specify the frequency using a Likert scale ranging from 1 to 4 (1 – occasionally; 2 – many times; 3 – frequently; 4 – very frequently) and severity of that symptom using a Likert scale ranging from 1 to 3 (1 - mild; 2 - moderate; and 3 – severe).^36^ In this study, Cronbach’s alpha was = .81.

#### Attachment

Attachment orientations were examined using the Experiences in Close Relationships Questionnaire – Relationship Structure (ECR-RS).^38^ The ECR-RS is a 9 items self-report scale that assesses attachment patterns in a variety of close relationships, in this case, the relationship between the informal caregiver and the person with dementia. It assesses attachment-related anxiety (3 items; item example: “I’m afraid that other people may abandon me”) and attachment-related avoidance (item example: “I find it easy to depend on others” – reversed). Items are scored on a 7-point Likert scale (1 = “strongly disagree” to 7 “strongly agree”). In the present study, the Cronbach alpha was .77 for anxiety and .89 for avoidance.

### Procedure

The study was approved by the Ethical Committee of the CIP-Universidade Autónoma de Lisboa and by the hospitals’ Clinical Research Ethics Committees. Data was collected between January 2019 and December 2021. Informal caregivers were approached by doctors or psychologists during routine consultations of the persons living with dementia they care for to inform them about the general objectives of the study. Interested participants were contacted by one of the researchers and were evaluated individually to assess their appropriateness for participation in the study. Written informed consent was gathered from participants (ie, informal caregivers and patients living with dementia when possible). After consent, participants attended an interview. Each interview lasted about 1:30/2 hours.

Regarding non-caregivers, they were recruited in the community through informal advertisement, using a snowball sampling approach between March and June 2019. They filled out paper-pencil questionnaires. The time required to complete the questionnaires varied from 7 to 10 minutes.

All participants were volunteers and received no incentives or monetary compensation for their participation in the study.

### Data Analysis

Statistical analyses were conducted in IBM-SPSS statistical software program (version 28). Descriptive statistics, internal consistency, and bivariate correlations were calculated for all study variables. Differences between caregivers and non-caregivers among study variables were calculated using chi-square test and Independent Sample t-tests.

Moderation analyses were conducted using the PROCESS macro model 1.^39^ NPS were included as predictors, attachment orientations (ie, attachment anxiety and attachment avoidance) as moderators, and ER (ie, expressive suppression and cognitive reappraisal) as outcomes. Interactions were probed by examining interaction significance and the predictive effect of each factor at different levels of the moderator (the mean, at −1 SD and +1 SD of the mean). Effect size of moderation analyses were examined using f2 effect size according to the following guidelines: >.02 small; >.15 medium; >.35 large.

## Results

### Participants

#### Informal Caregiver Sample (n = 78)

A total of 78 informal caregivers of persons living with dementia participated in this study. Their mean age was 64.84 years (SD = 13.32; Min = 19; Max = 89). Most of them were women and were married (72%). In terms of their relationships with the patient, most were the spouse (54%), followed by adult children (35%). In terms of education, 41% had basic education and 23% completed high school. Most of them were retired (60%) and around 13% were unemployed.

In terms of the persons living with dementia they cared, 56% were women. They had on average 77 years old (SD = 7.59; Min = 47; Max = 92). Regarding their education, most had primary education (70.1%), and were retired (96%). The most common type of dementia was Alzheimer’s disease (62.8%) followed by vascular dementia (7.7%), Lewy body dementia (5.1%), frontotemporal dementia (2.6%), and other (21.8%). On average, the diagnosis was made 5.52 years ago (SD = 4.34), ranging from 6 months to 19 years.

#### Non-caregiver sample (n = 84)

With respect to the 84 non-caregivers, their age ranged from 50 to 83 years (M = 61.07, SD = 7.50). About 74% (n = 62) were women, and about 61% were married. In terms of education, 40% had a university education, followed by those who completed high school (24.1%). More than half of the non-caregivers were employed (63%) and 29% were retired.

### Differential Analyses

There are no significant differences between informal caregivers and non-caregivers, regarding sex (Χ2 (1) = .08, *P* = .77), age (men, t (29.8) = 1.91, *P* = .07; women, t (86.04) = 1.37, *P* = .17), and marital status (Χ^2^ (3) = .341, *P* = .33). However, there were differences according to education (Χ^2^ (1) = 8.92, *P* < .01). Results from crosstabulation showed that there are 49 caregivers with 9 or less years of school, while there are 41 non-caregivers with the same education level. On the other hand, there are 33 non-caregivers with twelve or more years of education, while there are only 20 caregivers with the same education level. This suggests that non-caregivers are more likely to have a higher level of education than caregivers that are more likely to have a lower education.

Differences according to occupation status were also found (Χ^2^ (1) = 20.82, *P* < .001). Results from crosstabulation showed that there are 20 caregivers who are employed, while 55 are unemployed. On the other hand, there are 50 non-caregivers who are employed, while 29 are unemployed. Again, this suggests that non-caregivers are more likely to be employed than caregivers that are more likely to be retired.

Also, results showed that informal caregivers have significantly higher mean values of suppression and attachment anxiety. On the contrary, they presented significantly lower mean values of attachment avoidance (Table 1).

**Table 1.** Sociodemographic Characteristics and Descriptive Statistics of Study Variables for IC and Non-ICs.

Variable	Caregivers (n = 78)		Non-Caregivers (n = 84)		Difference
	M (SD)	%	M (SD)	%	
Sex					
Male		71.8		73.8	*X*^*2*^ (1) = .08
Female		28.2		26.2	
Education					
9 years or less		59.7		36.1	***X*** ^***2***^ (1) = 8.92**
12 years of more		40.3		63.9	
Marital status					
Married		71.8		60.7	*X*^*2*^ (3) = .341
Single/widow/divorced		28.2		39.3	
Occupation status					
Active		26.7		63.3	***X*** ^***2***^ (1) = 20.82***
Non-active		73.3		36.7	
ER_supression	4.55 (1.64)		3.65 (1.41)		***t***_**(160)**_ = 3.75***
ER_reappraisal	5.18 (1.20)		5.02 (1.39)		***t***_**(160)**_ **= .78**
Att_Avoidance	2.06 (1.59)		3.01 (1.82)		***t***_**(160)**_ = −3.52**
Att_anxiety	2.74 (1.28)		2.25 (.83)		***t***_**(130.84)**_ = 2.84**
NPS	45.15 (31.26)		-	-	***-***

Note. **P < .01; ***P < .001. Significant differences are flagged in bold; ER, emotion regulation; Att, attachment; NPS, neuropsychiatric symptoms. Non-active occupation status includes unemployed, retired, or domestic work without remuneration.

### Correlational Analyses

Variables were significantly correlated in the expected directions. NPS were positively associated with expressive suppression. Expressive suppression was positively associated with cognitive reappraisal. Attachment avoidance was negatively associated with attachment anxiety (see Table 2).

**Table 2.** Correlations Among NPS, ER, and Attachment in Caregivers (N = 78).

	NPS	ER_Suppression	ER_Reappraisal	Att_Avoidance	Att_Anxiety
NPS	-				
ER_Suppression	.316*	-			
RE_ Reappraisal	.090	.269*	-		
Att_Avoidance	.209	.117	−.046	-	
At_Anxiety	−.071	−.008	.080	−.273*	-

Note. *P < .05; ER, emotion regulation; Att, attachment.

### Interaction Analyses: The Role of NPS and Attachment

We investigated whether NPS, attachment anxiety/attachment avoidance, and the interaction between the 2 were significant predictors of expressive suppression and cognitive reappraisal. Results are presented in Table 3.

**Table 3.** Conditional Effects of Attachment Orientation in the Link Between NPS and Emotion Regulation.

	Coeff	SE	t	*P*	95% LLCI	95% ULCI
Model 1 – Attach avoidance and suppression						
Effect of NPS on suppression	.05	.01	3.95	<.001	.023	.072
Effect of attach avoidance on suppression	.62	.23	2.66	.010	.154	1.089
Interaction NPS* Attach avoidance	−.01	.00	−3.020	.004	−.020	−.004
	Effect	Boo SE			Boo 95% LL	Boo 95% UL
	Conditional effects at values of the moderator					
Low attach avoidance	.04	.01	3.96	<.001	.018	.054
Medium attach avoidance	.02	.01	3.28	.002	.009	.036
High attach avoidance	.00	.01	.50	.620	−.012	.020
	Coeff	SE	t	*P*	95% LLCI	95% ULCI
Model 2 attach avoidance and CR						
Effect of NPS on CR	.01	.01	.55	587	−.014	.025
Effect of attach avoidance on CR	−.11	.19	−.59	.560	−.486	.266
Interaction NPS* Attach avoidance	−.00	.00	−.06	.952	−.007	.006
Model 3 – Attach anxiety and suppression						
Effect of NPS on suppression	.03	.02	1.76	.084	−.004	.064
Effect of attach anxiety on suppression	.20	.27	.74	.460	−.342	.747
Interaction NPS* Attach anxiety	−.00	.01	−.79	.434	−.017	.008
Model 4 – Attach anxiety and CR						
Effect of NPS on CR	.01	.01	.89	.376	−.014	.037
Effect of attach anxiety on CR	.19	.21	.91	.369	−.225	.597
Interaction NPS* Attach anxiety	−.00	.00	−.65	.520	−.012	.006

Note. Coeff, coefficient; SE, standard error; LLCI, lower level of the 95% confidence intervals; ULCI, upper level of the 95% confidence intervals; Attach, attachment; CR, cognitive reappraisal.

#### Attachment Avoidance

**Figure 1.**
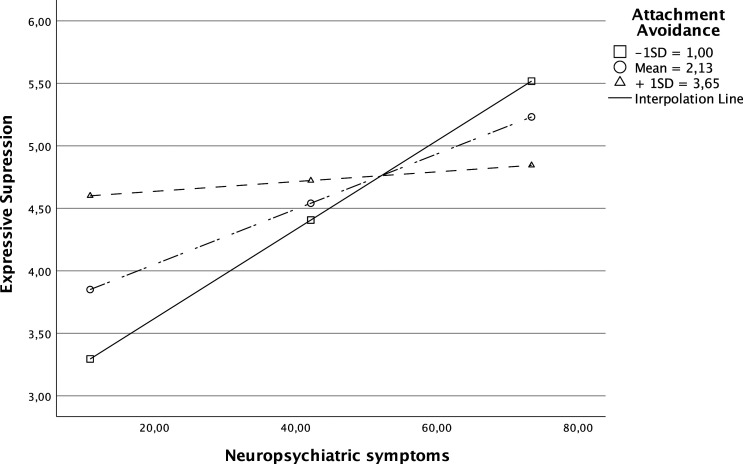
Attachment avoidance as moderator of the link between NPS and expressive suppression.

For expressive suppression, the model accounted for 22% of the variance in expressive suppression (R^2^ = .223, F = 5.55 (3, 58), *P* < .01). Both NPS (b = .05, *P* < .001) and attachment avoidance (b = .62, *P* = .010) were associated with more expressive suppression. The interaction between NPS and attachment avoidance was also significant (b = −.01, *P* = .004) and explained an additional 12% of the variance in expressive suppression. This interaction was probed by examining the association between NPS and expressive suppression when attachment avoidance was at the mean, and at −1 SD and +1 SD of the mean. NPS only predicted expressive suppression when attachment avoidance was 1 SD below the mean (b = .04, *P* < .001) or at the mean (b = .02, *P* = .002). When attachment avoidance was 1SD above the mean it did not predict expressive suppression (b = .00, *P* = .620) (see Figure 1). A medium effect size was found for this moderation result (f^2^ = .28).

For cognitive reappraisal, the model accounted for 2% of the variance in cognitive reappraisal (R^2^ = .029, F = .58 (3, 58), *P* = .634). Neither NPS (b = .01, *P* = .587) nor attachment avoidance (b = −.11, *P* = .560) were associated with cognitive reappraisal. The interaction between NPS and attachment avoidance was also non-significant (b = −.00, *P* = .952). A small effect size was found for this moderation result (f^2^ = .03).

#### Attachment Anxiety

For expressive suppression, the model accounted for 11% of the variance in expressive suppression (R^2^ = .11, F = 2.38 (3, 58), *P* = .079). Neither NPS (b = .03, *P* = .084) nor attachment anxiety (b = .20, *P* = .460) were associated with expressive suppression. The interaction between NPS and attachment anxiety was also non-significant (b = −.00, *P* = .434). A small effect size was found for this moderation results (f^2^ = .12).

A similar pattern was found for cognitive reappraisal. The model accounted for 2% of the variance in cognitive reappraisal (R^2^ = .02, F = .44 (3, 58), *P* = .725). Neither NPS (b = .01, *P* = .376) nor attachment anxiety (b = .19, *P* = .369) were associated with cognitive reappraisal. The interaction between NPS and attachment anxiety was also non-significant (b = −.00, *P* = .520). A small effect size was found for this moderation result (f^2^ = .02).

## Discussion

The aim of this study was to examine the role played by NPS and attachment in explaining individual differences in the way caregivers of persons living with dementia regulate their emotions. To the best of our knowledge, there are no studies assessing differences between caregivers and non-caregivers in the way they regulate their emotions.

While several studies have pointed to the existence of differences between caregivers and non-caregivers in terms of psychological distress, burden, and burnout, among others,^1-3^ none of them have examined differences in terms of ER. Results partially confirmed our H1. They suggested that caregivers of persons living with dementia seemed to suppress more their emotions than non-caregivers, but no differences were found for cognitive reappraisal. As expected, and found in other contexts,^16^ our results seem to suggest that caregivers are more likely to suppress their emotions. This may happen for several reasons. On one hand, by suppressing their emotions, especially when distressed or burdened, caregivers can maintain the appearance that they are emotionally adapted to the role and challenge of being a caregiver of persons living with dementia. On the other hand, and because ER is an interpersonal process,^26^ it is possible that deficits and declines in emotion behaviours of persons living with dementia diminish the interactions between the dyad and, consequently, the caregivers seem to inhibit more their emotional expression. However, more studies are needed to better understand if in other contexts of caregiving, that are not characterized by the existence of these deficits or declines, the pattern is similar. This is because participants were asked to report about the way they regulate their emotions in general and not specifically in the context of their relationships with the persons living with dementia. Regarding cognitive reappraisal, no differences were found between caregivers and non-caregivers. Indeed, because cognitive reappraisal is an ER strategy effective in reducing negative affect it is possible that individuals use this strategy across different contexts.

Additionally, while some studies exist about the role played by NPS and attachment orientations on the psychological functioning of caregivers (in terms of burden, quality of life, and physical health),^19-22^ none of these studies have examined if these variables were associated with caregivers’ ER. Because ER is an important factor in explaining individuals’ psychological functioning,^8-11^ it is of high importance to better understand how and why caregivers regulate their emotions in a specific way. Results partially confirmed our H2. As hypothesized, NPS and attachment avoidance were positively associated with expressive suppression. NPS have been linked to several outcomes of the caregivers,^19,21^ and our result suggested that they seem to play a role also in caregivers’ ER in terms of expressive suppression. As discussed before, deficits and declines in emotion behaviours of persons living with dementia may contribute to increasing caregivers’ expressive suppression. Also, expressive suppression may be a way of showing that they are well-adjusted and coping well with the caregiving task. Indeed, we did not know if in this context expressive suppression is useful since we did not evaluate its effects. As suggested for some studies in different contexts of caregiving, expressive suppression may be beneficial for maintaining the mental health of formal caregivers.^40^ The authors suggested that for some formal caregivers, venting emotions could lead to resentment and negative affect or could prevent them from performing caregiving tasks. It is possible that the same happened with informal caregivers. NPS, however, were not associated with caregivers’ ER in terms of cognitive reappraisal. This may be explained by the fact that cognitive reappraisal is less used when dealing with stressful events (such as care for an individual with dementia with NPS) since it is a demanding strategy difficult to implement.^41^

As expected, attachment avoidance was positively associated with expressive suppression as proposed by previous studies. More avoidantly attached individuals usually use deactivating strategies to inhibit or dampen their emotions to avoid feeling vulnerable and rejected or to avoid proximity to others.^29,33,34^ Attachment anxiety was linked neither to expressive suppression nor to cognitive reappraisal. Similar results have been found in other studies with some authors proposing that concerns about others’ availability may lead to complex effects on emotion processes according to the type of emotion experienced (see Brandão et al^42^ for further details).

Finally, our H3 about the moderating role of attachment in the link between NPS and ER, was also partially confirmed. Only attachment avoidance moderated the link between NPS and expressive suppression. NPS were only associated with expressive suppression for individuals with lower/middle levels of attachment avoidance. This is an interesting result since we had hypothesized the contrary. However, it is possible that more avoidantly attached individuals use expressive suppression as a regular strategy not being dependent on the presence of NPS within this context. For those with lower levels of attachment avoidance, expressive suppression may be used for dealing with the stress that results from the presence of NPS, usually associated with the relatives’ suffering. Additionally, research suggests that in close relationships, emotion expression can serve to seek support.^43,44^ However, this may not always be the case. Specifically, if an individual does not perceive their partner as capable of attending to their needs, they may be less inclined to express emotions that signal their need for support.

## Limitations and Future Research

Results from this study should be interpreted with caution. This is a cross-sectional study. Thus, inferences about causal associations between variables cannot be made. Second, the sample is small being composed mainly of women. Thus, future studies should explore the links between these variables using a larger and a more heterogenous sample.

Third, we did not evaluate the outcomes associated with the use of expressive suppression; thus, we did not know if the use of expressive suppression within this context is useful or not, especially in the presence of NPS. Also, the ERQ measures overall expressive suppression not only related to the caregiving context. Future studies should further explore the underlying motives for using expressive suppression and the effects that this strategy of ER has on their psychological functioning (and for the psychological and health functioning of persons living with dementia). More studies are needed about the use of cognitive reappraisal since no significant associations were found in this study.

### Clinical Implications

This study highlights the need of offering psychological support to caregivers that cope with NPS of their relatives living with dementia. While dependent on the motives underlying its use, expressive suppression usually has negative consequences (eg, increase blood pressure, disrupt communication, impact psychological well-being), and can compromise responsiveness to others.^15,45^ Therefore, it is recommended that caregivers have access to psychological interventions that specifically target ER (such as the intervention developed by Moskowitz et al, which focuses on positive emotion regulation)^46^ especially in the presence of NPS, and for those with lower/middle levels of attachment avoidance to promote caregivers’ resources to respond to their relative’s needs to maintaining a healthy psychological functioning.
